# Do Renal Cysts Affect the Success of Extracorporeal Shockwave Lithotripsy? A Retrospective Comparative Study

**DOI:** 10.1155/2013/978180

**Published:** 2013-06-06

**Authors:** Adnan Gücük, Ufuk Öztürk, Uğur Üyetürk, Eray Kemahlı, Güven Akın, M. Abdurrahim İmamoğlu, Ahmet Metin

**Affiliations:** ^1^Department of Urology, Abant Izzet Baysal University Faculty of Medicine, 14200 Bolu, Turkey; ^2^Department of Urology, Ministry of Health, Ankara Diskapi Yildirim Beyazit Education and Research Hospital, Ankara, Turkey

## Abstract

The aim of this study was to assess the effect of simple renal cysts on extracorporeal shockwave lithotripsy (SWL) in patients with calyceal renal calculi. Patients with simple renal cysts >35 mm and ipsilateral renal calculi <20 mm that were treated with SWL constituted group 1 (cyst + calculi). The control group included patients aged >40 years that had renal calculi <20 mm and no cysts that were treated with SWL. The 2 groups were compared according to age, gender, body mass index, calculi size, localization, and density, the calculi fragmentation rate, and the percentage of stone-free patients. Mean cyst size in group 1 was 44.04 ± 9.08 mm. Mean age in group 1 was 61.4 ± 10.2 years versus 56.9 ± 8.2 years in the control group; the difference was significant (*P* = 0.045). There were not any other significant differences between the 2 groups, except for the stone-free rate (*P* > 0.05), which was 33.3% in group 1 and 68.2% in the control group (*P* = 0.017). The presence of renal cysts in a patient with calculi requires that an individualized treatment plan be devised, so as to provide the patient with the most effective treatment.

## 1. Introduction

Simple renal cysts are nonhereditary benign cystic disorders that occur in 50% of patients aged >50 years. The prevalence of the cysts increases with age and the cysts increase in size—on average—by 2.8 mm each year [1]. Although they are benign, they can be symptomatic due to their size. Some anatomical anomalies that hinder urine outflow, such as cystic renal diseases, ureteropelvic stenosis, urethral stenosis, and diverticulum of the calyx, may change the treatment modalities in patients with renal calculi. 

Currently, extracorporeal shock wave lithotripsy (SWL) is the most common mode of therapy for small renal calculi. Calculi are first disintegrated by shock waves, and then the fragments are spontaneously cleared from the urinary tract [2]. Additional therapy is required if the calculi cannot be spontaneously cleared from the urinary tract, which increases the cost of treatment and causes the renal parenchyma to be exposed to unnecessary shock waves; therefore, it is of the utmost importance to identify which patients will benefit from SWL therapy. Any factor that obstructs the normal outflow of urine may cause urinary calculi formation. SWL is not contraindicated in patients with renal cysts and calculi; however, renal cysts may limit the success of SWL because of the pressure on and distortion of the collecting system they cause [3].

It was reported that SWL may be performed in patients with cysts and calculi without an increase in complications [3]. To the best of our knowledge, there are no comparative studies on SWL therapy in patients with simple renal cysts. As such, the present retrospective study aimed to evaluate the effect of renal cysts on SWL in patients with renal calculi. 

## 2. Materials and Methods

**Figure 1:**
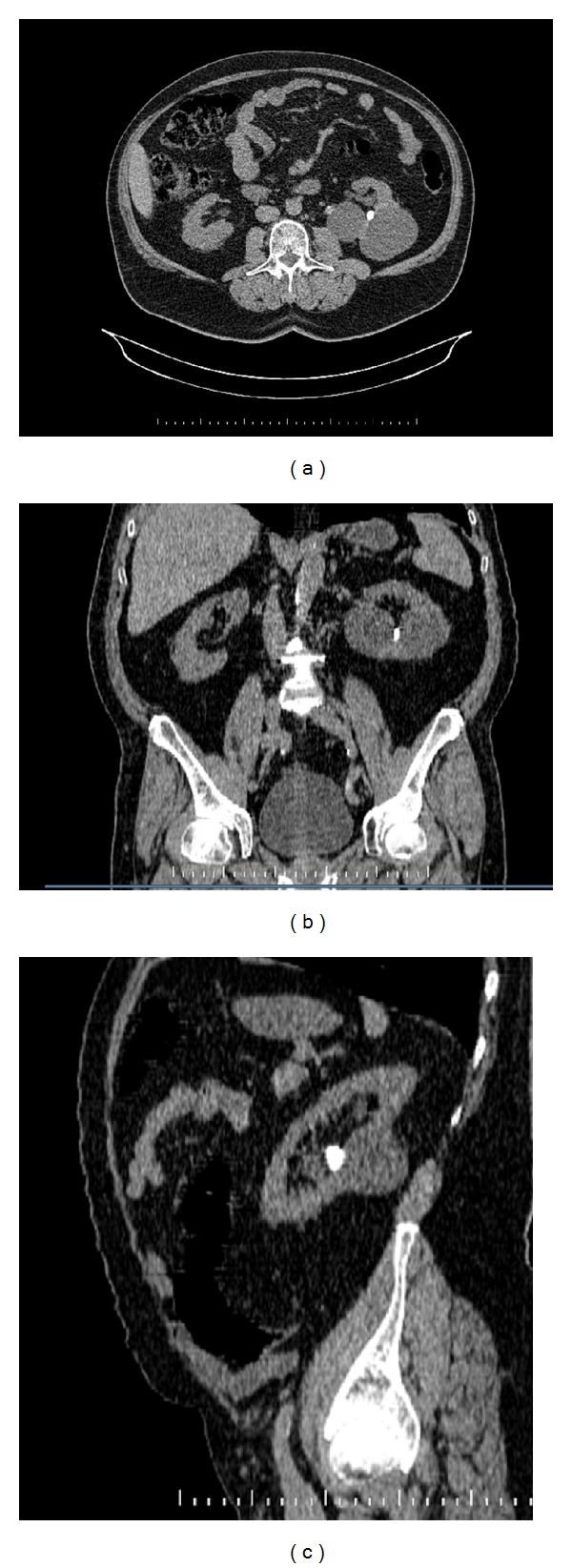
CT images of a patient with cysts and stone ((a) transvers, (b) coronal, and (c) sagittal).

Following institutional review board approval of the study protocol data for the patients that underwent SWL in our clinics between January 2007 and May 2011 were analyzed retrospectively. Patients with a simple renal cyst or cysts >35 mm and solitary ipsilateral renal calculi that had undergone SWL were included in group 1 (cyst + calculi) (Figure 1). The control group (group 2) included patients aged >40 years that had renal calculi <20 mm and no cysts that had undergone SWL. Patients with cysts other than Bosniak type 1 or 2 simple cysts, those with cysts other than simple cysts, and those with cysts <35 mm were excluded from the study. Patients with renal pelvis calculi, multiple calculi in different calyxes, and calculi >20 mm were also excluded. 

Mean age of the patients and calculi size and localization in group 1 were determined. To form a similar control group, SWL patients aged >40 years with calyx calculi <20 mm were selected in a consecutive, randomized fashion. The control group was twice the size of group 1. Patients underwent ultrasonography and computed tomography (CT, noncontrast- and contrast-enhanced) prior to SWL therapy. Cysts were measured via CT and the maximum diameters were recorded. Calculi density was recorded in Hounsfield units (HU) via non-contrast-enhanced CT, as previously described [4]. Calculi were measured according to their stone surface area, based on European Association of Urology guidelines [5]. Calculi localization was classified as lower pole and other (middle calyx and upper pole calyx). A Siemens Modularis Variostar lithotripter (Siemens AG, Medical Solutions, Erlangen, Germany) was used to perform SWL therapy until the calculi were fragmented or for a maximum of 3 treatment sessions. SWL was considered unsuccessful if after SWL treatment sessions there was no fragmentation or residual calculi >4 mm were observed on radiological examination (kidney-ureter-bladder radiography and renal ultrasonography) 3 months after treatment.

The 2 groups were compared on the basis of age, gender, body mass index (BMI), stone size, localization, and density, the stone fragmentation rate, and the stone-free rate. Statistical analysis was performed using SPSS v.15.0 software (SPSS Inc. Chicago, IL, USA). In addition to frequency and percentage distribution of the data, Student's *t*-test was used for between-group comparisons, and the chi-square test was used to determine differences between categorical data. Statistical significance was set at *P* < 0.05.

## 3. Results

The study included 65 patients; group 1 included 21 patients and the control group included 44 patients. Mean age in group 1 was 61.4 ± 10.2 years (range: 40–82 years) in group 1 versus 56.9  ±  8.2 years (range: 46–76 years) in the control group (*P* = 0.045). Mean cyst size in group 1 was 44.04  ±  9.08 mm (range: 35–69 mm). In 8 of the patients (38.1%) in group 1 the cysts were in the lower and middle poles, and in 13 patients (61.9%) they were located in the upper pole and middle calyx region.

There were not any differences in gender, BMI, stone size, the stone fragmentation rate, or HU values between the 2 groups (Table 1). Calculi were localized in the lower pole in 9 patients in group 1 (42.9%) and in 16 patients in the control group (36.4%); the difference was not significant (*P* = 0.818). In all, 7 patients in group 1 (33.3%) were stone-free versus 30 patients (68.2%) in the control group; the difference was significant (*P* = 0.017). Of the 7 stone-free patients in group 1, 2 had cysts in the lower pole and 5 had cysts in the upper pole. Other than temporary hematuria and the usual pain following SWL, there were no complications in either group.

## 4. Discussion

Cystic renal disorders do not cause metabolic alterations that result in calculi formation. The notion that anatomic anomalies that interfere with urine outflow may both increase calculi formation and decrease the clearance of calculi without causing metabolic alterations seems rational [6]. Chang et al. reported that 24% of patients with renal cysts had renal calculi and that 11.5% of patients without renal cysts had renal calculi [7]. The current opinion is that the presence of renal cysts or polycystic kidneys is not a contraindication for SWL [3]. Although renal cysts do not contraindicate SWL therapy, they may negatively affect its outcome once they begin to have an effect on calyceal anatomy. Previous studies on patients with renal cysts and calculi that received SWL therapy included patients with peripelvic, polycystic, or solitary simple cysts—none of which are homogeneous in nature [3, 6, 8]. To the best of our knowledge, the present study is the first to show that simple renal cysts negatively affect the outcome of SWL. 

Calculi size and localization and renal anatomy are equally important when examining the stone-free rate following SWL therapy as are calculi density and type and BMI [9]. El-Nahas et al. reported that it is difficult to discern calculi composition prior to SWL therapy, and even if known it would be insufficient for estimating calculi response to therapy [9]. They reported that evaluation based on non-contrast-enhanced CT prior to SWL is more effective for estimating the response to SWL than knowing the composition of calculi. They also reported that a high BMI (>30) and calculi density >1000 HU negatively affect calculi fragmentation [9]. In the present study there were not any significant differences in calculi size and localization, BMI, or stone density between the groups (*P* < 0.05). 

Mean age in group 1 was significantly higher than in the control group (*P* = 0.045). Even though we selected patients aged >40 years to ensure that the control group would be similar in age, this result was expectable keeping in mind that cystic diseases are seen in advanced age groups. Philippou et al. investigated the relationship between SWL and patient age and reported that there was not a significant difference in the fragmentation or stone-free rates between the patients aged >70 years and those aged <70 years [10]. For these reasons, we claim that the difference in age between the groups in our study did not affect the outcomes. 

Data concerning the presence of renal cysts in patients with renal calculi and their effect on SWL therapy are limited. Cass reported a 43% stone-free rate in 13 patients with renal cysts and calculi 3 months after SWL [8]. Deliveliotis et al. achieved a 100% fragmentation rate and a 60% stone-free rate in 15 patients [3]. Studies that included nonhomogeneous small groups had a 25% SWL treatment success rate in polycystic patients versus 72% in patients with simple renal cysts [11–13]. Furthermore, they achieved a success rate of 60% in patients with multiple cysts and 83% in those with solitary cysts and reported that cysts affected the stone-free rates [13]. It is known that simple renal cysts do not disperse SWL shock waves or alter their effectiveness; as such, they do not interfere with calculi fragmentation [14–16]. In the present study there were not any differences in calculi fragmentation between group 1 and the control group. The stone-free rate in the present study was 33.3% in group 1 and 68.2% in the control group, and the difference was significant. There were not any differences between the groups in terms of fragmentation, but there was a significant difference in calculi cleaning, which might have been due to the fact that cysts have a negative effect the cleansing of the residual calculi. 

Most studies on SWL and patients with renal cysts and calculi were conducted to assess the effect of SWL on cysts [8]. In some studies SWL was performed because of the presence of renal cysts, and hemorrhaging in the cysts was reported. Bleeding in cysts was reported as an incidental side effect of SWL that is not problematic in patients with cysts that were previously asymptomatic and infection-free [3]. In the present study cystic hemorrhage was not observed during radiological followup 3 months after SWL therapy. Of course, not all renal cysts cause distortion in the calyxes and lead to SWL failure, which largely depends on cyst size and localization. A large cyst that is thoroughly cortical will not cause any disturbances; however, a smaller cyst localized in the parenchyma or in the parapelvic region may cause problems. As we could not find any data concerning the relationship between cyst size and calyceal distortion in the literature, we evaluated patients with cysts >35 mm, but if this relationship is established by larger trials in the future, more dependable and specific suggestions can be made for the relationship of the size of the cyst and SWL treatment. In patients with calculi and cysts that cause calyceal distortion, we do not think SWL therapy should be the first-line treatment option, but instead we recommend percutaneous aspiration + sclerotherapy or minimally invasive methods, such as laparoscopic cyst decortication; it is more efficacious to administer SWL following such procedures. In cases where cystic distortion does not hinder the passage of flexible ureterorenoscope, retrograde renal surgery could also be an alternative treatment.

The present study has some limitations, including a small patient population and the lack of a prospective randomized design. In order to prove that the distortion of the cyst is the factor that prevents the calculi cleansing in these patients, the calculi's outcome should be followed up after the cyst treatment through a procedure such as percutaneous or laparoscopy. Prospective studies in which these shortcomings are removed may present more scientific data.

## 5. Conclusion

While considering the treatment options in patients with renal calculi, the presence of cysts should be kept in mind. Although calculi fragmentation is achieved in such patients, the stone-free rate may be lower than in other patients, which is why treatment in such patients should be individualized. In patients with cysts that cause calyceal distortion initially treating the cyst and then considering SWL therapy may be a viable option; in this manner optimum effectiveness of SWL therapy can be provided for this patient group.

## Figures and Tables

**Table 1:** Data of the patients.

Characteristic	Group 1 (cyst + calculi)	Control group (calculi only)	*P* value
Mean age (years)	61.47 ± 10.2	56.95 ± 8.2	0.045*
(range)	(40–82)	(46–76)	
Gender (male/female)	15/6	29/15	0.872
Mean BMI (kg/m²)	28.07 ± 4.09	28.5 ± 3.82	0.643
(range)	(21.41−35.42)	(22.41–36.54)	
Mean stone surface area (mm²)	112 ± 76.74	106.02 ± 44.02	0.528
(range)	(42–296)	(45–220)	
Mean stone density (HU value)	798 ± 256.1	708.5 ± 214.3	0.627
(range)	(468–1286)	(420–1320)	
Stone fragmentation rate	20/21 (95.2%)	43/44 (97.7%)	0.754
Stone-free rate	7 (33.3%)	30 (68.2%)	0.017*

*Significant *P* value.

## References

[1] Terada N, Ichioka K, Matsuta Y, Okubo K, Yoshimura K, Arai Y (2002). The natural history of simple renal cysts. *Journal of Urology*.

[2] Paik ML, Resnick MI (2000). Is there a role for open stone surgery?. *Urologic Clinics of North America*.

[3] Deliveliotis C, Argiropoulos V, Varkarakis J, Albanis S, Skolarikos A (2002). Extracorporeal shock wave lithotripsy produces a lower stone-free rate in patients with stones and renal cysts. *International Journal of Urology*.

[4] Gücük A, Uyetürk U, Oztürk U, Kemahlı E, Yıldız M, Metin A (2012). Does the hounsfield unit value determined by computed tomography predict the outcome of percutaneous nephrolithotomy?. *Journal of Endourology*.

[5] Tiselius H-G, Andersson A (2003). Stone burden in an average Swedish population of stone formers requiring active stone removal: how can the stone size be estimated in the clinical routine?. *European Urology*.

[6] Amar AD, Das S, Egan RM (1981). Management of urinary calculous disease in patients with renal cysts: review of 12 years of experience in 18 patients. *Journal of Urology*.

[7] Chang C-C, Kuo J-Y, Chan W-L, Chen K-K, Chang LS (2007). Prevalence and clinical characteristics of simple renal cyst. *Journal of the Chinese Medical Association*.

[8] Cass AS, Preminger GM (1995). Extracorporeal shock wave lithotripsy for renal stones with renal cysts present. *Journal of Urology*.

[9] El-Nahas AR, El-Assmy AM, Mansour O, Sheir KZ (2007). A prospective multivariate analysis of factors predicting stone disintegration by extracorporeal shock wave lithotripsy: the value of high-resolution noncontrast computed tomography. *European Urology*.

[10] Philippou P, Lamrani D, Moraitis K, Bach C, Masood J, Buchholz N (2012). Is shock wave lithotripsy efficient for the elderly stone formers? Results of a matched-pair analysis. *Urological Research*.

[11] Psihramis KE, Dretler SP (1987). Extracorporeal shock wave lithotripsy of caliceal diverticula calculi. *Journal of Urology*.

[12] Fuchs G, Chaussy C (1988). Patient selection for extracorporeal shock wave lithotripsy. *Difficult Diagnosis in Urology*.

[13] Cass AS, Lee JY, Aliabadi H (1992). Extracorporeal shock wave lithotripsy and endoscopic management of renal calculi with urinary diversions. *Journal of Urology*.

[14] Lingéman JE, McAteer JA, Gnessin E, Evan AP (2009). Shock wave lithotripsy: advances in technology and technique. *Nature Reviews Urology*.

[15] Delakas D, Daskalopoulos G, Cranidis A (1997). Extracorporeal shockwave lithotripsy for urinary calculi in autosomal dominant polycystic kidney disease. *Journal of Endourology*.

[16] Gambaro G, Fabris A, Puliatta D, Lupo A (2006). Lithiasis in cystic kidney disease and malformations of the urinary tract. *Urological Research*.

